# Application of Spatial Risk Assessment Integrated With a Mobile App in Fighting Against the Introduction of African Swine Fever in Pig Farms in Thailand: Development Study

**DOI:** 10.2196/34279

**Published:** 2022-05-31

**Authors:** Weerapong Thanapongtharm, Vilaiporn Wongphruksasoong, Waratida Sangrat, Kittin Thongsrimoung, Nattavut Ratanavanichrojn, Suwicha Kasemsuwan, Amnat Khamsiriwatchara, Jaranit Kaewkungwal, Kansuda Leelahapongsathon

**Affiliations:** 1 Department of Livestock Development Bangkok Thailand; 2 Faculty of Veterinary Medicine Kasetsart University Nakhon Pathom Thailand; 3 Center of Excellence for Biomedical and Public Health Informatics (BIOPHICS), Faculty of Tropical Medicine, Mahidol University Bangkok Thailand

**Keywords:** African swine fever, multi-criteria decision analysis, risk-based surveillance, risk assessment, spatial analysis

## Abstract

**Background:**

African swine fever (ASF), a highly contagious disease affecting both domestic and wild pigs, has been having a serious impact on the swine industry worldwide. This important transboundary animal disease can be spread by animals and ticks via direct transmission and by contaminated feed and fomites via indirect transmission because of the high environmental resistance of the ASF virus. Thus, the prevention of the introduction of ASF to areas free of ASF is essential. After an outbreak was reported in China, intensive import policies and biosecurity measures were implemented to prevent the introduction of ASF to pig farms in Thailand.

**Objective:**

Enhancing prevention and control, this study aims to identify the potential areas for ASF introduction and transmission in Thailand, develop a tool for farm assessment of ASF risk introduction focusing on smallholders, and develop a spatial analysis tool that is easily used by local officers for disease prevention and control planning.

**Methods:**

We applied a multi-criteria decision analysis approach with spatial and farm assessment and integrated the outputs with the necessary spatial layers to develop a spatial analysis on a web-based platform.

**Results:**

The map that referred to potential areas for ASF introduction and transmission was derived from 6 spatial risk factors; namely, the distance to the port, which had the highest relative importance, followed by the distance to the border, the number of pig farms using swill feeding, the density of small pig farms (<50 heads), the number of pigs moving in the area, and the distance to the slaughterhouse. The possible transmission areas were divided into 5 levels (very low, low, medium, high, and very high) at the subdistrict level, with 27 subdistricts in 10 provinces having very high suitability and 560 subdistricts in 34 provinces having high suitability. At the farm level, 17 biosecurity practices considered as useful and practical for smallholders were selected and developed on a mobile app platform. The outputs from the previous steps integrated with necessary geographic information system layers were added to a spatial analysis web-based platform.

**Conclusions:**

The tools developed in this study have been complemented with other strategies to fight against the introduction of ASF to pig farms in the country. The areas showing high and very high risk for disease introduction and transmission were applied for spatial information planning, for example, intensive surveillance, strict animal movement, and public awareness. In addition, farms with low biosecurity were improved in these areas, and the risk assessment developed on a mobile app in this study helped enhance this matter. The spatial analysis on a web-based platform helped facilitate disease prevention planning for the authorities.

## Introduction

### Background

African swine fever (ASF) is a highly contagious disease affecting both domestic and wild pigs of all ages. It is highly contagious because the morbidity and mortality in exposed pig herds are possibly up to 100% [1]. The ASF virus (ASFV) is a large, enveloped, double-stranded DNA virus with a size of 170 to 190 kbp, and its genome includes >50 structural proteins and several nonstructural proteins [2,3]. Owing to the large size and complex structure of the virus, there is no effective vaccine producible at the moment [2]. The virus is also highly survivable, allowing the virus to spread via various sources, including infected live and dead pigs, infected pork products, contaminated feed, and contaminated fomites [3]. The ASFV can survive for up to 18 months in serum at room temperature and for several months in raw pork products such as raw ham or sausage and treated products [2]. It survives for a longer time in frozen material and resists a pH level between 4 and 13 [4]. Hence, it is a serious disease in the swine industry worldwide, causing serious economic and production losses [3].

The World Organization for Animal Health identifies ASF as an important transboundary animal disease that has spread across almost all continents and in many countries [3]. After the ASF genotype I was first detected in Kenya in 1910, it circulated in several countries in Africa [1]. The virus was then introduced to Europe, where it was first found in Portugal in 1957 and spread to many countries in Europe and also in South and Central America [3,5,6]. Besides Sardinia [7], ASF type I has been successfully eradicated in Europe and America [1,2]. Subsequently, ASF genotype II emerged in Africa and was first introduced to Europe in 2007 and spread to many countries, beginning with Georgia [8] to its neighbors [5,6] and across to the west [9]. The virus was first introduced to Asia in 2018 in China and, since then, the disease has spread to many countries in Asia and the Pacific [10]. In 2019, 11 countries encountered the ASF, including Mongolia, Vietnam, Cambodia, Hong Kong, the Democratic People’s Republic of Korea, Laos, Myanmar, the Philippines, the Republic of Korea, East Timor, and Indonesia. In 2020, 2 more countries were affected by the disease: Papua New Guinea and India. The latest outbreak was detected in the north of east Malaysia in 2021 on the Borneo island, which it shares with Brunei and Indonesia [9].

The transmission cycle of ASF worldwide includes 3 main hosts: wild pigs, soft ticks, and domestic pigs. The ASFV can replicate in the soft ticks of the genus Ornithodoros, which are mainly found in Africa and some parts of Europe [11]. This type of tick has been identified as a biological vector of the ASFV and has spread the virus to wild and domestic pigs [1,11]. Pig-to-pig transmission occurs via direct and indirect contact. Direct contact between infected and susceptible pigs has been identified as a very effective transmission route [12]. Indirect contact shows that the ASFV is introduced to susceptible pigs through people, contaminated feed, infected boar semen, and contaminated fomites [11,13]. Experimental studies have found that the Stomoxys flies can be a mechanical vector transmitting ASFV to domestic pigs for a limited time [14,15]; however, ASFV tested in flies collected on ASF-affected farms in Lithuania produced negative results [11]. Feeding pigs with contaminated pork products or fodder is considered to play a major role in the transmission of ASF across countries. Although the import of pigs and pork products from ASF-infected countries was officially banned, it was found that the first outbreak in Europe occurred in a pig farm near the Lisbon airport in Portugal in 1957 caused by feeding pigs waste from airline flights [16]. The same occurred in 2007; the ASF genotype II was first introduced to Georgia through contaminated pork carried by international ships that was then fed to pigs [8].

ASF has a high impact not only on the commercial pig industry but also on smallholders. The introduction of ASF to countries has resulted in many impacts, for example, the loss of up to 50% of the pig population, affecting food security, the cost of disease control, and the loss of status for international trade [16]. The greatest losses occur in countries where most pig farmers are smallholders or practice backyard farming [16]. This sector usually relates to low farm biosecurity, poor knowledge of disease prevention, and a lack of financial resources for farm improvement [17-20]. Europe has experience in ASF spread and successful eradication, the lessons learned including, for example, that (1) pig holders with poor biosecurity usually facilitate the first occurrence of the outbreak [16]; (2) in the areas dominated by commercial pig production, strict animal movement and implementation of culling policies successfully prevented the spread of the disease [16]; and (3) in endemic areas mostly dominated by poor biosecurity farming, apart from both aforementioned measures, the eradication program emphasized improving farm biosecurity, increased disease awareness in pig farmers, and extensive monitoring activities [16].

### Objectives

Southeast Asia, where Thailand is located, has been facing the spread of ASF [10]. Immediately after ASF was reported in China in 2018 [21], Thailand has been intensively preventing the introduction of ASF to pig farms in the country by implementing the control measures learned from other countries, in particular European countries [5,6,16,22]. We conducted this study to enhance the measures for preventing the introduction of the ASFV to high-risk farms, focusing on high-risk areas in the country, as well as for assisting responsible officers in spatial information planning. Therefore, the objectives of this study were 3-fold: (1) to identify the potential areas for ASF introduction and transmission in Thailand, (2) to develop a tool for farm assessment of ASF risk introduction focusing on smallholders, and (3) to develop a spatial analysis tool that is easily used by local officers for disease control planning.

## Methods

We developed tools for ASF prevention and control, including (1) a suitability map for ASF introduction and transmission in the first stage of virus introduction; (2) a mobile app for farm assessment of ASF risk introduction; and (3) a web application for spatial analysis of ASF prevention and control by combining a layer of suitability map, locations with risk level of farm assessment, and other relevant layers. The methods for each step are detailed in the following sections.

### Developing a Suitability Map for ASF Introduction and Transmission

We applied a knowledge-driven model called a spatial multi-criteria decision analysis (MCDA) to determine the suitability areas for ASF introduction and transmission. The analytical hierarchy process, one of the MCDA methods, was used in this study for its power and simplicity [23]. The analysis consisted of four steps: (1) defining and standardizing risk factors, (2) assigning relative importance to the risk factors, (3) combining all layers of risk factors, and (4) assessing the sensitivity and uncertainty of the analysis.

We used a participatory approach [24] by inviting 20 experts in relevant fields, including 12 (60%) epidemiologists, 2 (10%) virologists, and 6 (30%) stakeholders in pig production, to define, standardize, and assign the relative importance of the risk factors of ASF introduction and transmission in the country, with emphasis on the first stage of virus introduction. Each expert initially assigned individual outputs, and then all experts assigned the final outputs together. The defined factors were standardized using fuzzy membership functions [25] in which the relationship between the values of each factor and the suitability for ASF introduction and transmission ranging from 0 (unsuitable) to 1 (highly suitable) was defined. There were 4 types of relationships proposed to the experts—namely, linear, sigmoidal (s-shaped), j-shaped, and user-defined—with increasing, decreasing, or symmetrical functions [25]. The Fuzzy tool from the IDRISI software (Clark Labs) [26] was used to implement this standardization step. The Fuzzy tool requires the position along the x-axis of each risk factor of 4 parameters (*a*, *b*, *c*, and *d*) governing the shape of the fuzzy membership function [25].

A pairwise comparison technique was used to define the relative importance of each factor. The procedure consisted of comparing each pair of factors using a 9-point continuous comparison scale (Table 1). The weight value for each factor (*W_i_*) was calculated by taking the eigenvector corresponding to the largest eigenvalue of the pairwise score matrix and then normalizing the sum of the components to a unity [27-29]. The consistency ratio (CR), which is calculated as the consistency index divided by a random index, was used to verify the consistency of the matrix. The random index, derived from the study by Saaty [30], depends on the number of analyzed factors (3 factors=0.58, 4 factors=0.90, 5 factors=1.12, 6 factors=1.24, 7 factors=1.32, 8 factors=1.41, 9 factors=1.46, 10 factors=1.49, 11 factors=1.51, 12 factors=1.54, 13 factors=1.56, 14 factors=1.57, and 15 factors=1.58). The consistency index is calculated as



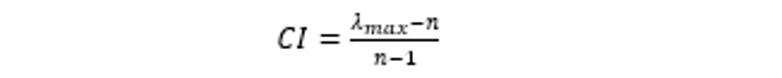



where *λ*_max_ is the maximum eigenvalue of the judgment matrix and *n* is the number of factors. If the CR is >0.10, then some pairwise values need to be reconsidered, and the process is repeated until the desired value of CR of <0.10 is reached [30].

**Table 1:** The 9-point scale values used in the pairwise comparison of factors.

Intensity of importance	Description
1	Equal importance
3	Moderate importance
5	Strong or essential importance
7	Very strong or demonstrated importance
9	Extreme importance
2, 4, 6, 8	Intermediate values
Reciprocals	Values for inverse comparison

The suitability map was produced by incorporating all standardized factor layers using the weighted linear combination (WLC) [31] method in the R software (R Foundation for Statistical Computing). The packages *raster*, *maptools*, and *fields* in R were used. In the WLC, each standardized factor is multiplied by its corresponding weight, these are summed, and then the sum is divided by the number of factors. Its equation is as follows: 



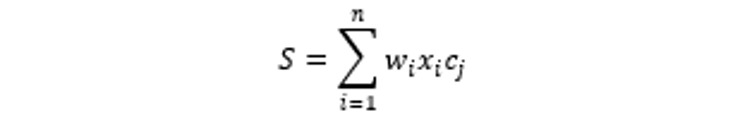



where *w_i_* is the weight of criterion *i*, *x_i_* is the criterion score of criterion *i* (value of the corresponding raster cell in the criterion raster map), *n* is the number of criteria, and *c_j_* is the criterion score (1 or 0) of constraint *j*.

With regard to the sensitivity analysis, we applied the one-at-a-time method, which works by changing 1 input factor at a time and evaluating the effect of the change on the output [32]. It was selected for its simplicity and good comparability results. The sensitivity analysis was carried out for each factor by setting 2 parameters: a step size of 1% and a range of 50% (–25% to +25%) [33]. By changing 1 factor at a time, all other factors can be fixed, at least to a great extent, to their central or baseline value. The sum of all criteria weights at any percent change (PC) level should always be equal to 1. The weight of the main changing criterion (*W*(*c_m_*, *pc*)) at a certain PC level can be calculated as follows: *W*(*cm*, *pc*) = *W*(*cm*, *0*) + (*W*(*cm*, *0*) * *pc*), 1≤*m*≤*n*, where *W*(*c_m_*, 0) is the weight of the main changing criterion *c_m_* at the base run (the original weights). The weights of the other criteria *W*(*c_i_*, *pc*) are adjusted proportionally in accordance with *W*(*c_m_*, *pc*) to maintain the sum of all criteria weights at any PC of 1 with the following equation:



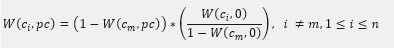



where *W*(*c_i_*, 0) is the weight of the *i*th criterion *c_i_* at the base run.

We evaluated this step using the mean of the absolute change rate (MACR) [34]. In each simulation, the original suitability map (the original weights) and the output map of the alternative model (changing criterion weights) were quantitatively matched through a pixel-by-pixel comparison. The MACR was calculated using the following equation:



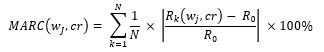



where *MARC*(*w_J_*, *cr*) is the mean absolute value of the change rate, with *w_J_* as the change rate, and *N* is the number of pixels. In addition, an uncertainty surface resulting from the changes in weights was produced for the study area representing the SD of the different suitability maps [35,36].

The spatial data used in this part are listed in Table 2. The distance risk factors were processed using the cost distance tool in ArcGIS (version 10.2; Esri) [37], in which the objects from the nearest distances, including the border, the ports, and the slaughterhouses, were estimated. Pig movement data in 2018 were obtained from a web-based movement registration system [38] (e-Movement), through which the movements of pigs and other animals are required to be registered. Pig population data were obtained from the animal census data operated by the Department of Livestock Development (DLD) officers annually, in which pig farms with <50 pigs were included for analysis. Surveys of pig farms using swill feeding were conducted by local DLD officers between September 2018 and December 2018. All geographical data were converted into raster data sets with a 100-meter resolution using ArcGIS.

**Table 2:** Spatial risk factors, standardized methods, and relative importance of each factor.

Spatial risk factors	Fuzzy membership functions	Inflection points					Weights
		a	b	c	d		
Distance to border, m	Sigmoidal monotonically decreasing	10,000	10,000	10,000	100,000	0.2295	
Distance to port, m	Sigmoidal monotonically decreasing	10,000	10,000	10,000	100,000	0.3567	
Distance to slaughterhouse, m	Sigmoidal monotonically decreasing	10,000	10,000	10,000	100,000	0.0580	
Pigs moving in the area, n	Sigmoidal monotonically increasing	1	5	5	5	0.0668	
Density of small pig farms (<50 heads), farms/km^2^	Sigmoidal monotonically increasing	0.1	1	1	1	0.1219	
Pig farms using swill feeding, farms/km^2^	Sigmoidal monotonically increasing	0.01	0.5	0.5	0.5	0.1670	

### Developing a Mobile App for Farm Assessment of ASF Risk Introduction

We also applied the analytical hierarchy process to develop a set of factors and algorithms for risk assessment, which was performed through a platform in a mobile app. Publications on ASF risk factors were reviewed and proposed to the experts, who then selected and standardized the factors. First, risk factors for ASF introduction at the farm level such as biosecurity measures and characteristics of the farms’ environments were selected by the experts. The selected factors were also standardized by defining the relationship between each of the factors and suitability using a 5-point scale (1=very low, 2=low, 3=medium, 4=high, and 5=very high). The relative importance of each factor was then defined using a pairwise comparison technique.

We designed questionnaires and coded algorithms on a mobile app for both iOS and Android operating systems by applying the outputs obtained from the previous steps. The combination of all factors to produce a final weighted estimate of suitability was implemented using a mobile app. The final score of each farm was obtained from the WLC method categorized into 5 suitability levels (<1.5=very low, >1.5-2.5=low, >2.5-3.5=medium, >3.5-4.5=high, and >4.5=very high). We set the provinces that found high and very high suitability for ASF distribution areas (analyzed in the previous step) as the targeted areas for farm assessment. District livestock officers were trained on how to install and use the app and then evaluated all non–Good Agricultural Practice pig farms in their responsible areas between April 2019 and July 2019.

### Developing a Spatial Analysis of ASF Prevention and Control on a Web Application

We developed a spatial analysis of ASF prevention and control on a web-based platform. The necessary geodata for prevention and control planning were provided, including a suitability map for ASF introduction and transmission (step 1), the farm locations with ASF risk level (step 2), and other relevant layers such as locations of slaughterhouses (collected by DLD staff). The buffer rings surrounding selected pig farms were also developed, which allows users to download the important data in spreadsheet files, such as the number and details of neighboring farms and the distance to selected farms.

### Ethics Approval

This study was approved by the Research Committee of the Bureau of Disease Control and Veterinary Services, DLD, Thailand (permit 64(2)-0105-110).

## Results

### A Suitability Map for ASF Introduction and Transmission

**Figure 1:**
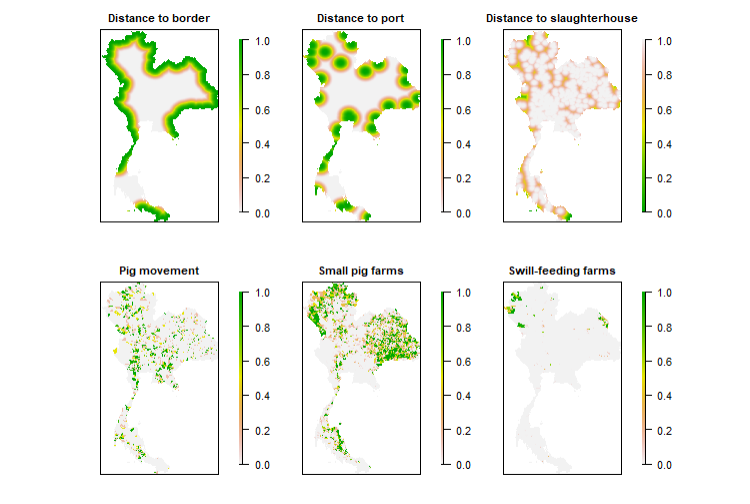
Maps of the standardized risk factors used to analyze the suitability for African swine fever introduction and transmission in Thailand. From top left to bottom right: the distance to the border, the distance to the port, the distance to the slaughterhouse, the number of pigs moving in the area, the density of small pig farms (<50 heads), and the number of pig farms using swill feeding.

Table 2 shows the results of the defined factors, the standardized methods, and the relative importance of each factor, where distances are measured in meters and areas are measured in square kilometers (km^2^). Six spatial risk factors were identified by the experts: (1) the distance to the border, (2) the distance to the port, (3) the distance to the slaughterhouse, (4) the number of pigs moving in the area, (5) the density of small pig farms (<50 heads), and (6) the number of pig farms using swill feeding. The results showed that, according to the experts, the distance to the port had the highest weight, followed by the distance to the border, the number of pig farms using swill feeding, the density of small pig farms (<50 heads), the number of pigs moving in the area, and the distance to the slaughterhouse. Figure 1 shows the standardized risk factors used to produce the final suitability map for ASF distribution.

**Figure 2:**
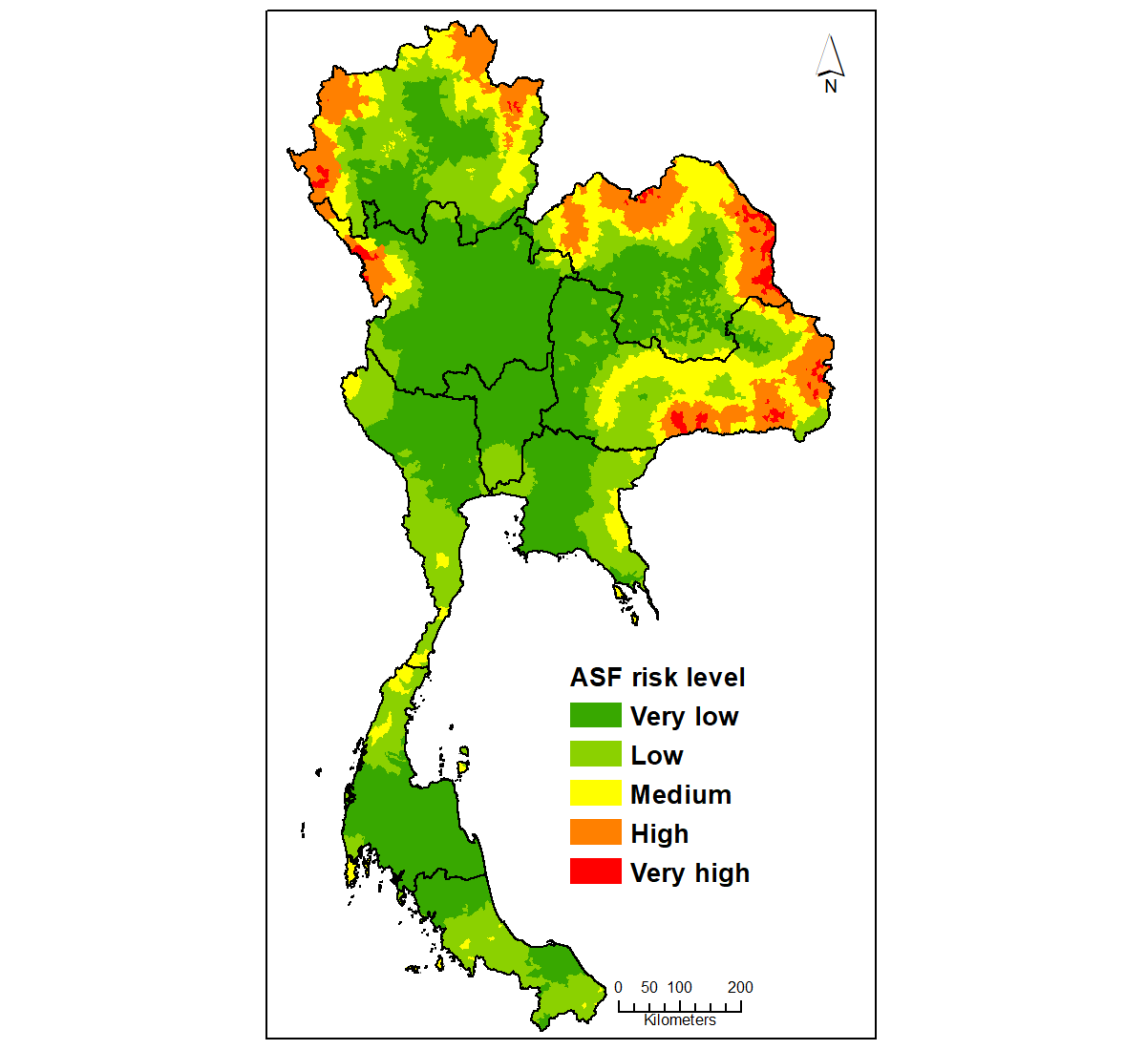
The suitability map for African swine fever (ASF) introduction and transmission in Thailand.

Figure 2 shows the suitability map for ASF introduction and transmission in Thailand if it were first introduced to the country. The resulting most potential areas were clustered near the north and northeast borders. The entire area was extracted and aggregated into 5 levels (very low to very high) at the subdistrict level, as shown in Table 3. There were 27 subdistricts in 10 provinces with very high suitability and 560 subdistricts in 34 provinces with high suitability.

**Table 3:** Number of subdistricts, districts, and provinces according to African swine fever (ASF) risk levels.

ASF risk levels	Subdistricts (N=7416), n (%)	Districts (N=926), n (%)	Provinces (N=77), n (%)
Very high	27 (0.4)	17 (1.8)	10 (12.9)
High	560 (7.6)	144 (15.6)	34 (44.2)
Medium	1408 (18.9)	353 (38.1)	52 (67.5)
Low	2693 (36.3)	490 (52.9)	75 (97.4)
Very low	5833 (78.7)	478 (51.6)	55 (71.4)

**Figure 3:**
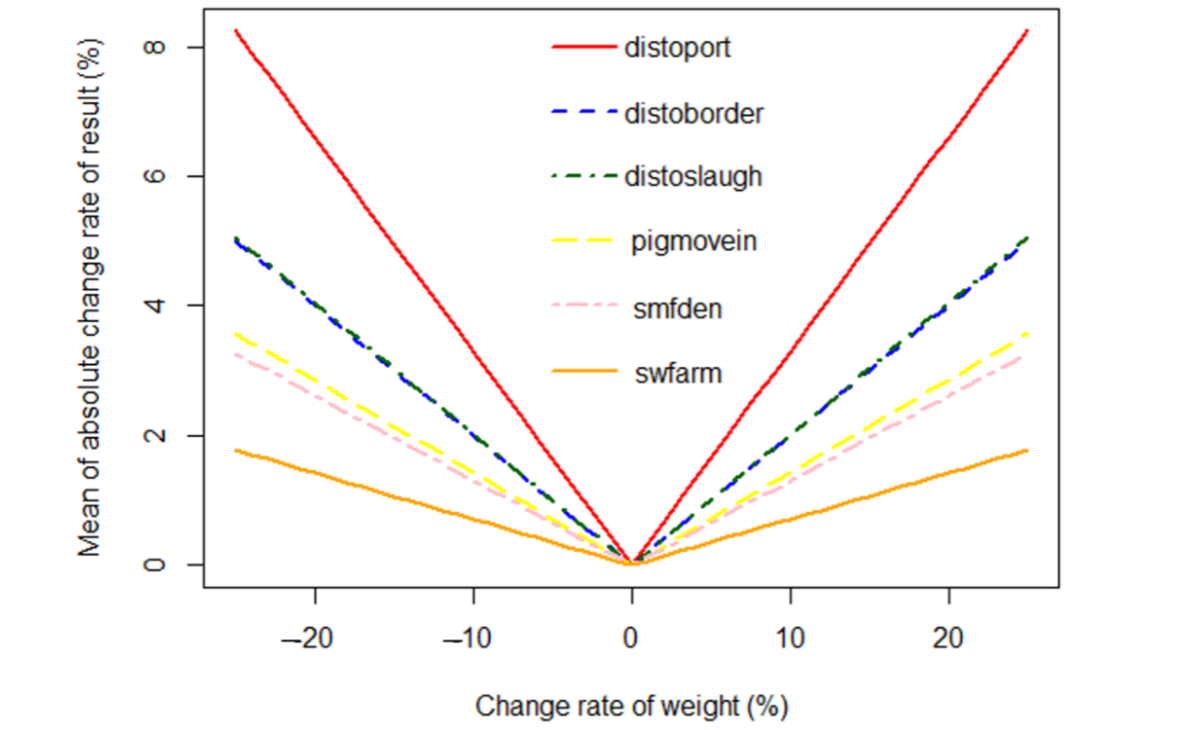
The results of the one-at-a-time sensitivity analysis. Distoborder: the distance to the border; distoport: the distance to the port; distoslaugh: the distance to the slaughterhouse; pigmovein: the number of pigs moving in the area; smfden: the density of small pig farms; swfarm: the number of pig farms using swill feeding.

Figure 3 shows the results of the one-at-a-time sensitivity analysis, in which the simulated suitability maps for ASFV transmission in pigs in Thailand were generated with the weight of each factor changed from −25% to 25% with a step size of 1%. The MACRs were used to display the sensitivity of each factor, with a high gradient indicating a greater change in the values of the output maps (high sensitivity). It appeared that the most sensitive factor was the distance to the port followed by the distance to the border, the distance to the slaughterhouse, the number of pigs moving in the area, the density of small pig farms, and the number of pig farms using swill feeding.

**Figure 4:**
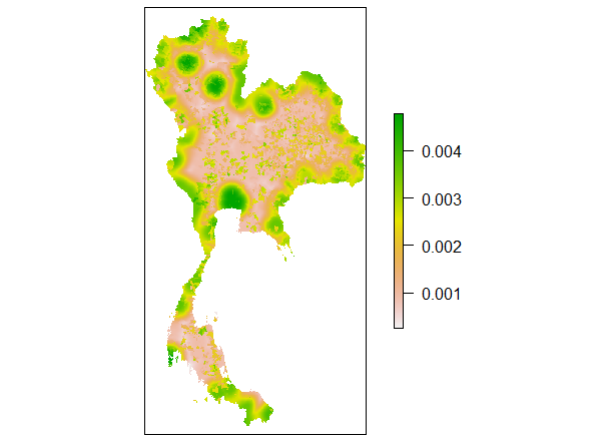
Uncertainty map: SD of the suitability maps for African swine fever introduction and transmission in pigs in Thailand.

The uncertainty analysis showed fairly robust results and a spatial heterogeneity. The uncertainty surface remained stable, with the maximum SD value being <0.1 (Figure 4) even though the risk factors were varied. This implies that the predicted suitability areas for ASFV transmission in pigs in Thailand according to the suitability index are fairly robust.

### Farm Assessment of ASF Risk Introduction Using a Mobile App

The defined risk factors of ASF introduced to pig farms, standardized risk factors, and relative importance of risk factors (weight) are shown in Multimedia Appendix 1 [39-54]. The experts defined 17 risk factors that would be important at the farm level, which were categorized into 3 groups: farm biosecurity, farm management, and farm location. The 3 most important identified factors were the pig feed, the farm location on an ASF risk level, and the breeding practices on the farm.

The results of the farm assessment of ASF introduction are presented in Table 4. There were 61,747 pig farms in 34 provinces evaluated using the developed app on a mobile platform. Of the 61,747 evaluated farms, 4 (0%) and 4380 (7.09%) were found to have very high and high risk of ASF introduction, respectively.

**Table 4:** The results of pig farms assessed using an app developed on a mobile platform.

Risk assessment level	Farms (N=61,747), n (%)	Provinces (N=34), n (%)
Very low	3919 (6.4)	32 (94.1)
Low	23,604 (38.2)	33 (97.1)
Medium	29,840 (48.3)	32 (94.1)
High	4380 (7.1)	28 (82.4)
Very high	4 (0)	4 (11.8)

### A Spatial Analysis of ASF Prevention and Control on a Web Application

**Figure 5:**
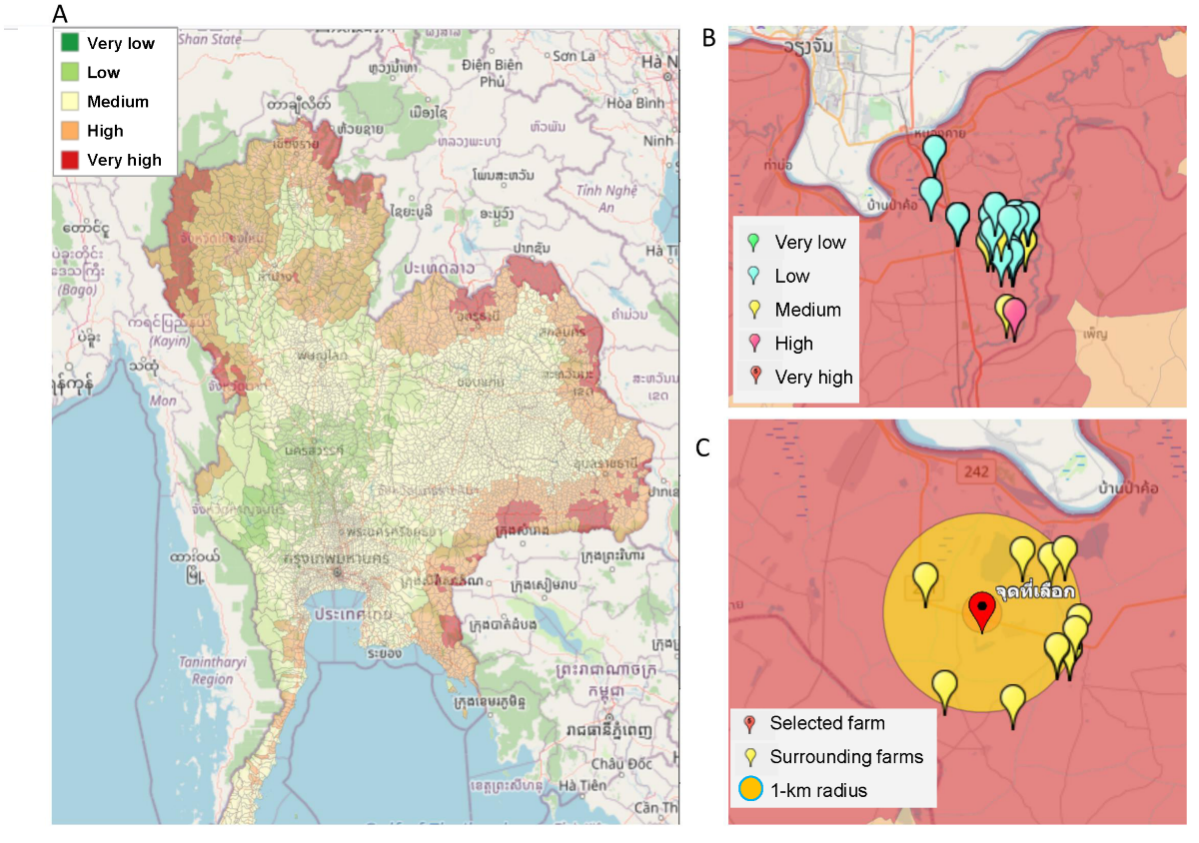
A spatial analysis of African swine fever (ASF) prevention and control on a web application. A spatial analysis conducted by integrating all relevant layers for ASF prevention and control, such as (A) an ASF risk map, (B) farm locations with ASF risk at the farm level, and (C) the buffer zones and farms surrounding a selected farm.

Figure 5 shows a spatial analysis on a web application in which the components are composed of the outputs obtained from the previous processes, including the suitability map (step 1) and the locations of pig farms with risk-assessed level (step 2). In addition, we included other important layers useful for disease control planning, including the locations of the slaughterhouses.

## Discussion

### Principal Findings

The prevention of the introduction of ASF to Thailand started with the questions of how and where. As learned from the infected countries, the ASFV was introduced to the country possibly through infected live and dead pigs, infected pork products, infected boar semen, contaminated feed, and contaminated fomites [55,56]. Banning pigs, pork products, boar semen, animal feeds, and feed ingredients from affected countries; strictly checking pork products carried by travelers from affected countries; and encouraging pig farmers not to feed pigs with swill were immediately implemented by the Thai authorities [32]. However, with the increasing efficiency of virus detection and prevention, specific areas with a high possibility of virus introduction should be more focused on disease prevention activities [57,58]. Hence, a geographic information system–based MCDA was used in this study to evaluate the suitability for ASFV introduction and transmission in pigs in Thailand in the absence of actual data on ASFV occurrence in the country [59-61], where the outputs of the MCDA were required to help convert the current state of knowledge into a visualization. MCDA is a static, knowledge-driven model that ranks the best choices of a set of weighted rules based on existing publications and expert knowledge [61]. However, the quality of the predictions may be compromised by the misidentification of some unknown factors. It has been applied in many countries to predict suitability maps for ASF [59] and other animal diseases [24,36,62,63].

The 6 spatial risk factors identified and prioritized by the experts were the distance to the port, which had the highest weight; the distance to the border; the number of pig farms using swill feeding; the density of small pig farms (<50 heads); the number of pigs moving in the area; and the distance to the slaughterhouse. This consideration was based on the risk pathways of ASFV transmission by separating the factors into 3 groups in the offensive line. First, if the ASFV were introduced to the country through various sources [1,2,16], as a first line, it would officially and unofficially pass through international and border ports as well as border lines through smuggling [16,58]. Therefore, the distance to the port and to the border was initially included in the analysis. Second, if the ASFV passed through the first line to the territory by not being detected, as a second line, it would initially occur in pig farms that fed pigs with swill feed [16] or it would attack smallholders with poor farm biosecurity, as shown in Europe [16,64,65]. Finally, if the ASFV infected local pig farms, pig movement would facilitate the spread [16], and the virus would be present in slaughterhouses and adjacent areas [49,66].

Prevention of ASFV introduction to pig farms requires good farm biosecurity [16,67]. Biosecurity comprises the measures aimed at reducing the risk of introduction and spread of disease agents or, more simply, “to keep disease agents away from pigs” or “to keep pigs away from disease agents” [68]. Biosecurity practiced by large-scale commercial pig farms mostly corresponds to large investments in infrastructure and equipment that are hardly implemented by smallholders. However, biosecurity improvements in the smallholder sector can already be achieved through very simple and low-cost precautionary measures [69]. This study was focused on selecting biosecurity that is practical for smallholders (as described in Multimedia Appendix 1) and using a simple way for local officers or farmers to be able to evaluate pig farms through a mobile app platform. Risk management [58] can also be communicated through the app, in which biosecurity practices with low scores would be suggested for improvement. Moreover, the outputs of the spatial risk analysis part were added to the farm evaluation part, allowing farmers to be more concerned with biosecurity improvement if their farms are located in high-risk areas [70].

This study developed a spatial analysis web-based platform that can facilitate disease prevention and outbreak control implemented by the responsible officers. Spatial epidemiological analysis plays an important role in planning for disease prevention and outbreak control [61] and has been used to describe and visualize the spatial distribution of hosts [71,72] and diseases [73,74], identify clusters of diseases [73,75], and predict disease risk [36,62]. The outputs of the analyses can be applied for disease prevention [16,59,76], for example, conducting intensive surveillance in high-risk areas, mitigating the risk of disease transmission in high-risk areas by strict animal movement, improving biosecurity, and minimizing the number of susceptible hosts. Spatial analysis is also applied for outbreak control. As guided by the World Organization for Animal Health [77], following the confirmation of an outbreak, control areas based on epidemiological factors may be established around the affected premises. Control measures basically include restriction of animal movement, intensive surveillance, and other specific measures applied to the affected premises. Implementation of these activities requires knowledge of the extent of these areas, the number of animals and farms within the areas, the exact locations of the farms, and the exact locations for setting checkpoints. However, working on spatial analysis is limited by things such as computers with high capacity, geographic information system software, geodata, technicians, and time-consuming processes [61].

Although MCDA is a fast and easy approach to be applied for developing tools for risk assessment at the spatial and farm levels, the limitations may be caused by the approach itself. Knowledge-driven models such as MCDA provide an interesting alternative to model the suitability for ASFV distribution in space or at the farm level as a way to prioritize surveillance and improve prevention [78], but the quality of the predictions may be compromised by the misidentification of some unknown factors. For instance, the spatial risk factors used in this study were focused on the pig-to-pig transmission cycle; therefore, the outputs may not be suitable for the sylvatic transmission cycle as analyzed in Africa [59]. Regarding farm evaluation, the accuracy of the evaluation may cause doubt and needs to be further tested.

### Conclusions

This study developed tools by integrating a spatial risk assessment, a farm assessment on a mobile app, and a spatial analysis on a web-based platform aiming for the prevention of ASFV introduction to pig farms in Thailand. The high-risk areas of ASF transmission in Thailand extracted from a risk map were used for disease prevention, such as intensive surveillance, strict movement control, biosecurity improvement if possible or not raising pigs in the farm if not possible, and public awareness. The risk assessments developed on a mobile app were used to evaluate pig farms focusing on smallholders in the most prioritized areas based on a spatial risk assessment. A spatial analysis on a web-based platform was used by local authorities for spatial planning of disease prevention and could be used for outbreak control if an outbreak occurred. The tools developed in this study have been complemented with other strategies to fight against the introduction of the ASFV to pig farms in the country.

## Appendix

Multimedia Appendix 1 The defined risk factors of African swine fever introduced to farms, standardized factors, and weight of each factor.

## Abbreviations

ASF: African swine fever
ASFV: African swine fever virus
CR: consistency ratio
DLD: Department of Livestock Development
MACR: mean of the absolute change rate
MCDA: multi-criteria decision analysis
PC: percent change
WLC: weighted linear combination

## Data Availability

The data that support the findings of this study are available from the Department of Livestock Development, but restrictions apply to the availability of these data, which were used under license for this study and so are not publicly available. Data are available from the authors upon reasonable request and with permission from the Department of Livestock Development.
