# Supplementary material for: Application of Spatial Risk Assessment Integrated With a Mobile App in Fighting Against the Introduction of African Swine Fever in Pig Farms in Thailand: Development Study
Source: JMIR Form Res. 2022 May 31;6(5):e34279. doi: 10.2196/34279 (PMC9198819; doi:10.2196/34279)
Supplement: Multimedia Appendix 1 [file formative_v6i5e34279_app1.docx]

**Multimedia Appendix1.** The defined risk factors of African swine fever (ASF) introduced to farms, standardized factors, and weight of each factor.

| Factors | Suitability level (score of suitability level) | | | | | Weight |
| --- | --- | --- | --- | --- | --- | --- |
|  | Very low (1) | Low (2) | Medium (3) | High (4) | Very high (5) |  |
| - Certified standard farm [38,39] | Certified GAP^a^ farm | Used to be certified GAP but expired | Certified GFM^b^ farm | Used to be certified GFM but expired | Not certified | 0.0298 |
| - Type of pig house [40-44] | Close house (evaporation or wind tunnel) | Open house (enclosure walls and bird netting) | Open house (enclosure walls without bird netting) | Open house (without enclosure walls and bird netting) | Free raising | 0.0569 |
| - Animal husbandry area [45,46]   - separate animal and human areas   - having a fence effectively prevents other animals | Both | —^c^ | — | Only 1 | None | 0.0249 |
| - Management of new pigs before entering the farm or herd [47] | All in, all out | Quarantine new animals for at least 14 days | Quarantine new animals for <14 days | — | No quarantine of new animals | 0.0408 |
| - Other animals on the farm (select >1 choice) [44] | No other animals | Only poultry | — | Only dogs and cats | — | 0.0211 |
| - Management of vehicles before entering the farm [47,48] | Only use farm vehicles | Outside vehicles enter the farm area but are always disinfected before | — | Outside vehicles enter the farm area but are not always disinfected before | Outside vehicles enter the farm area but are not disinfected before | 0.0733 |
| - Management of people before entering the farm (bath, change of clothes, change of shoes, and disinfection) [38,47] | Perform all | 3 of 4 | 2 of 4 | 1 of 4 | None | 0.0702 |
| - Pig feed [41,49] | Only commercial feed or raw materials | — | Use swill feed boiling at least 30 minutes before | — | Use swill feed without boiling at least 30 minutes before | 0.1390 |
| - Human food (select >1) [40,48]   - buying pork for cooking on the farm   - water from the household flowing into the animal husbandry area   - cooking or consuming food in the animal-raising area | None | — | 1 choice | 2 choices | 3 choices | 0.0843 |
| - Source of drinking water for pigs (select >1) [44] | Tap water or disinfected water | Ground water or rain water | Well water | — | Water from natural sources | 0.0253 |
| - Breeding practices on the farm [44] | No breeding | Only use breeder or semen inside farm | Only use a certified breeding service | Use certified and uncertified breeding services | Use uncertified breeding service | 0.0812 |
| - Insect and parasite control [41,47] | Regular program | — | Have it but not usual | — | No program | 0.0083 |
| - Source of new coming pigs [41,47,50] | No new pig coming for more than a year | Certified farm and tested before | Uncertified farm but tested before | Certified farm but not tested before | Uncertified farm and not tested before | 0.0574 |
| - Management of pig selling [46] | Special place outside farm | — | Owner moving pigs to front of pen | — | Outside people catching pig in the pen | 0.0548 |
| - Management of pig carcass [46,47] | Burn or bury in the farm area | Consumed within the farm | Consumed outside the farm | — | Sold to merchant | 0.0393 |
| - Management of equipment (pig cages, food, and food sacks) and disinfection [44,47,48] | Do not share equipment with other farms | — | Share equipment but with disinfection | — | Share equipment without disinfection | 0.0631 |
| - Location of farm on an ASF risk level [41,47,48,51-53] | Very low | Low | Medium | High | Very high | 0.1303 |

^a^GAP: Good Agricultural Practice

^b^GFM: Good Farming Management

^c^Not available information

**References**

38. Asambe A, Sackey AKB, Tekdek LB. Sanitary measures in piggeries, awareness, and risk factors of African swine fever in Benue State, Nigeria. Trop Anim Health Prod. 2019;51:997–1001.

39. Martínez M, de la Torre A, Sánchez-Vizcaíno JM, Bellini S. 10. Biosecurity measures against African swine fever in domestic pigs. In: Iacolina L, Penrith M-L, Bellini S, Chenais E, Jori F, Montoya M, et al., editors. Understanding and combatting African Swine Fever. The Netherlands: Wageningen Academic Publishers; 2021. p. 263–81. doi:10.3920/978-90-8686-910-7_10.

40. Kabuuka T, Kasaija PD, Mulindwa H, Shittu A, Bastos ADS, Fasina FO. Drivers and risk factors for circulating African swine fever virus in Uganda, 2012–2013. Res Vet Sci. 2014;97:218–25.

41. Nantima N, Ocaido M, Ouma E, Davies J, Dione M, Okoth E, et al. Risk factors associated with occurrence of African swine fever outbreaks in smallholder pig farms in four districts along the Uganda-Kenya border. Trop Anim Health Prod. 2015;47:589–95.

42. Kukielka EA, Jori F, Martínez-López B, Chenais E, Masembe C, Chavernac D, et al. Wild and Domestic Pig Interactions at the Wildlife–Livestock Interface of Murchison Falls National Park, Uganda, and the Potential Association with African Swine Fever Outbreaks. Front Vet Sci. 2016;3. doi:10.3389/fvets.2016.00031.

43. Laddomada A, Rolesu S, Loi F, Cappai S, Oggiano A, Madrau MP, et al. Surveillance and control of African Swine Fever in free‐ranging pigs in Sardinia. Transbound Emerg Dis. 2019;66:1114–9.

44. Fasina FO, Kissinga H, Mlowe F, Mshang’a S, Matogo B, Mrema A, et al. Drivers, Risk Factors and Dynamics of African Swine Fever Outbreaks, Southern Highlands, Tanzania. Pathogens. 2020;9:155.

45. European Food Safety Authority. Evaluation of possible mitigation measures to prevent introduction and spread of African swine fever virus through wild boar. EFSA J. 2014;12. doi:10.2903/j.efsa.2014.3616.

46. Dione M, Ouma E, Opio F, Kawuma B, Pezo D. Qualitative analysis of the risks and practices associated with the spread of African swine fever within the smallholder pig value chains in Uganda. Prev Vet Med. 2016;135:102–12.

47. Bellini S, Rutili D, Guberti V. Preventive measures aimed at minimizing the risk of African swine fever virus spread in pig farming systems. Acta Vet Scand. 2016;58:82.

48. Fasina FO, Agbaje M, Ajani FL, Talabi OA, Lazarus DD, Gallardo C, et al. Risk factors for farm-level African swine fever infection in major pig-producing areas in Nigeria, 1997–2011. Prev Vet Med. 2012;107:65–75.

49. Heilmann M, Lkhagvasuren A, Adyasuren T, Khishgee B, Bold B, Ankhanbaatar U, et al. African Swine Fever in Mongolia: Course of the Epidemic and Applied Control Measures. Vet Sci. 2020;7:24.

50. Kouakou KV, Michaud V, Biego HG, Gnabro HPG, Kouakou AV, Mossoun AM, et al. African and classical swine fever situation in Ivory-Coast and neighboring countries, 2008–2013. Acta Trop. 2017;166:241–8.

51. Gulenkin VM, Korennoy FI, Karaulov AK, Dudnikov SA. Cartographical analysis of African swine fever outbreaks in the territory of the Russian Federation and computer modeling of the basic reproduction ratio. Prev Vet Med. 2011;102:167–74.

52. Oganesyan AS, Petrova ON, Korennoy FI, Bardina NS, Gogin AE, Dudnikov SA. African swine fever in the Russian Federation: Spatio-temporal analysis and epidemiological overview. Virus Res. 2013;173:204–11.

53. Martínez-López B, Perez AM, Feliziani F, Rolesu S, Mur L, Sánchez-Vizcaíno JM. Evaluation of the risk factors contributing to the African swine fever occurrence in Sardinia, Italy. Front Microbiol. 2015;06. doi:10.3389/fmicb.2015.00314.
